# The interaction between practice and performance pressure on the planning and control of fast target directed movement

**DOI:** 10.1007/s00426-016-0791-0

**Published:** 2016-08-17

**Authors:** Jonathan E. Allsop, Gavin P. Lawrence, Robert Gray, Michael A. Khan

**Affiliations:** 1 Vision and Eye Research Unit (VERU), Postgraduate Medical Institute, Anglia Ruskin University, Cambridge, UK; 2 School of Sport, Health and Exercise Sciences, Institute for the Psychology of Elite Performance, Bangor University, George Building, Holyhead Road, Bangor, Gwynedd LL57 2PZ UK; 3 Arizona State University, Phoenix, USA; 4 Faculty of Human Kinetics, University of Windsor, Windsor, Canada

## Abstract

Pressure to perform often results in decrements to both outcome accuracy and the kinematics of motor skills. Furthermore, this pressure–performance relationship is moderated by the amount of accumulated practice or the experience of the performer. However, the interactive effects of performance pressure and practice on the underlying processes of motor skills are far from clear. Movement execution involves both an offline pre-planning process and an online control process. The present experiment aimed to investigate the interaction between pressure and practice on these two motor control processes. Two groups of participants (control and pressure; *N* = 12 and 12, respectively) practiced a video aiming amplitude task and were transferred to either a non-pressure (control group) or a pressure condition (pressure group) both early and late in practice. Results revealed similar accuracy and movement kinematics between the control and pressure groups at early transfer. However, at late transfer, the introduction of pressure was associated with increased performance compared to control conditions. Analysis of kinematic variability throughout the movement suggested that the performance increase was due to participants adopting strategies to improve movement planning in response to pressure reducing the effectiveness of the online control system.

## Introduction

Perceived pressure to perform arises from both internal (i.e., heightened levels of state and personal performance expectations) and external factors (i.e., social evaluation and monetary rewards) and can be reliably indicated from the level and direction of anxiety associated with that same performance (e.g., state anxiety) (Gucciardi, Longbottom, Jackson, & Dimmock, 2010). The effect that this pressure has on sensorimotor performance has attracted significant research interest across domains ranging from surgery (e.g. Malhotra, Poolton, Wilson, Ngo, & Masters, 2012) to sport (e.g. Hardy, Beattie, & Woodman, 2007). In sport, the impairment of motor skills under pressure is termed ‘choking’ and defined as suboptimal performance in a situation of personal importance with strong incentives for accomplishment (Baumeister, 1984). However, detailed investigations into exactly which components of motor control are affected by pressure have yet to be fully explored (Lawrence, Khan, & Hardy, 2012b). Thus, the present study investigated how both the planning and control of movement change as a result of performance pressure.

Masters’ (1992) reinvestment theory, or conscious processing hypothesis (CPH), has gained significant research interest (e.g. Mullen & Hardy, 2000; Mullen, Hardy, & Tattersall, 2005) and states that pressure increases state anxiety and self-awareness about performing the skill successfully. This, in turn, causes performers to ‘reinvest’ (during the motor output) in previously developed rules about performing the skill in an attempt to control the mechanics of the movement (Masters & Maxwell, 2004). Since this is deemed important early in learning (Anderson, 1982; Fitts & Posner, 1967), the additional attention on the mechanics of the movement can lead to an increase in performance. Conversely, in the latter stages of learning, performance is deemed likely to deteriorate under conditions of increased state anxiety because the increase in skill focused attention and subsequent reinvestment leads to the breakdown of *normally* automatic processes (Gray, 2004).

Alternative explanations for the effects of pressure on performance can be found in distraction theories whereby task-irrelevant cues, such as state anxiety, compete with task-relevant information for limited cognitive resources (Eysenck, Deraksham, Santos, & Calvo, 2007; Wine, 1971). For example, attentional control theory (ACT; Eysenck et al., 2007) proposes that cognitive anxiety occupies processing and storage space of working memory, leading to a decrease in available task resources and potential decreases in performance. An increase in task effort may maintain or enhance performance, but the extra effort invested results in reduced processing efficiency (i.e., the relationship between performance and the amount of effort invested).

Whilst both ACT and CPH have received significant empirical support (e.g., Baumeister & Showers, 1986; Beilock & Carr, 2001; Gray, 2004; Langer & Imberm, 1979; Lawrence et al., 2012b; Lewis & Linder, 1997; Masters, 1992; Mullen & Hardy, 2000; Mullen et al., 2005; Wilson, Smith, & Holmes, 2007), this body of evidence has primarily focused on outcome measures of performance and is therefore limited in its ability to determine what affect pressure has on the underlying pre-planning and online control processes that lead to movement outcome.

Within the field of motor control, the notion that voluntary movement consists of both pre-planning and online control phases dates back to the nineteenth century (Woodworth, 1889) and has become the cornerstone of human target directed motor behaviour (see Elliott, Helsen, & Chua, 2001; Elliott et al., 2010 for reviews). The planning system has the goal of selecting and initiating a motor program based on the environmental and task demands of the situation, along with the positions of the performer’s body (Glover, 2004), and depends on feedforward processes involving discrepancies between predicted and actual sensory consequences (Desmurget & Grafton, 2000; Wolpert, Miall, & Kawato, 1998). The online control process is responsible for monitoring and adjusting the limb trajectories during the execution of the movement. These adjustments may be needed to reduce spatial errors in the movement execution caused by changes to the target, erroneous planning of the movement, and/or noise in the neuromotor system (Desmurget, Pélisson, Rossetti, & Prablanc, 1998).

Planning processes are said to involve a degree of conscious control (Klatzky, McCloskey, Doherty, Pellegrino, & Smith, 1987; Klatzky, Pellegrino, McCloskey, & Doherty, 1989), and are thus open to the influence of cognitive factors (Glover & Dixon, 2002; Glover, Rosenbaum, Graham, & Dixon, 2004). As such, pressure to perform and the processes within ACT could influence preplanning, whereby the cognitive (state) anxiety that arises from perceived pressure occupies a portion of working memory space and thus competes for resources that are needed for offline/pre-planning processes. Because online processes are said to be reflexive and attention-free in nature (Briere & Proteau, 2011; Proteau, Roujoula, & Messier, 2009; Veyrat-Masson, Briere, & Proteau, 2010), they lie outside of working memory and thus are less likely to be disrupted by the processes proposed within ACT. That is, the cognitive resources required for online control are significantly less than those of pre-planning and are therefore not likely to be affected by shifts to worrying thoughts and/or a reduction in one’s ability to inhibit these shifts. Whilst we propose that ACT cannot explain negative impacts to the online control phase of motor control, this is not the case for the CPH. Here, the presence of pressure to perform and the subsequent conscious attention directed to automatic processes (Briere & Proteau, 2011) would lead to a decrement in performance during movement execution.

Recently, Lawrence et al. (2012b) investigated the relationship between pressure on the online and offline processes movement. Participants performed aiming movements with both distance and direction accuracy requirements. The variability of limb trajectory kinematic profiles was calculated from the within-subject standard deviation at the distance travelled at peak acceleration (p*k*a), peak velocity (p*k*a), peak negative acceleration (p*k*na) and movement end (end) (see Khan et al., 2006 for a review). The rationale here was that if movements are programmed and not altered online then variability should increase as the movement progresses. This is because errors that occur early in the movement trajectory will be magnified as the movement distance increases. If however, corrections for variations in the movement trajectory are made during movement execution, then variability profiles would deviate from those that describe movement which is programmed in advance and not modulated online (Khan & Lawrence, 2005; Khan, Lawrence, Franks, & Elliott, 2003; Khan et al., 2003; Lawrence, Khan, Buckolz, & Oldham, 2006; Lawrence, Khan, Mourton, & Bernier, 2011; Lawrence, Gottwald, Khan, & Kramer, 2012a). Based on this analysis, Lawrence et al. (2012b) provided evidence that the presence of pressure to perform disrupted the use of the online movement adjustments in aiming tasks. Since online adjustments are reported to be reflexive in nature and outside of conscious control, Lawrence et al. (2012b) concluded that it is the processes proposed within the CPH (rather than ACT) that negatively impacted online correction processes eventually leading to choking in motor tasks.

Although the experiments of Lawrence et al. (2012b) helped to fill the research lacuna surrounding the effects of pressure on motor programming and control processes, the pressure manipulation was administered after only 90 acquisition trials and thus did not allow investigation into the effects of practice/skill level on this pressure–performance and motor control relationship. As previously stated, self-focus theories suggest the effects of pressure to perform differ depending on the stage of learning. Therefore, the present study aimed to more rigorously test the effect that pressure has on the preplanning and error correction phases of goal-directed movements both early *and* late in learning.

To achieve this, participants were asked to perform upper limb aiming movements under normal (low pressure) conditions and were transferred to high pressured conditions after both 30 (early in learning) and 400 (late in learning) practice trials. To investigate the effects of this pressure to perform transfer phases on offline and offline processes, the aforementioned variability methodology was adopted with profiles compared between the low and high pressure phases. It was hypothesised that pressure would affect performance based on a combination of processes underlying both CPH and ACT. Specifically, according to ACT it was expected that changes to preplanning would occur since these processes are dependent on working memory (Glover & Dixon, 2002; Glover et al., 2004). These effects would be revealed by differences in spatial variability at early kinematic markers when pressure is induced. Because online error-correction process are said to be automatic, attention-free, and lie outside of working memory (Briere & Proteau, 2011; Proteau et al., 2009; Veyrat-Masson et al., 2010; Lawrence et al., 2012b), we hypothesised that ACT cannot account for changes to these processes under pressure situations. However, according to CPH, it was expected that the presence of pressure to perform and the subsequent conscious attention to the automatic, attention-free online control would lead to a decrement in performance.

In specific regards to the early and late transfer to pressure, it was hypothesised that early in learning the introduction of pressure would be beneficial to performance since novices may actually benefit from the increased skill-focused attention caused by perceived pressure to perform. Any performance improvement would be supported by a decrease in spatial variability at later kinematic markers (i.e., increased online control of movement). Counter to this, because the task difficulty is low there may be limited subcomponents of movement execution to which to attend (Hill, Hanton, Mathews, & Flemming, 2010). Therefore, it is possible that performance would be impaired due to the anxiety that arises from pressure occupying working memory resources required for pre-planning (i.e., processes within ACT) leading to an increase in spatial variability at early kinematic markers (i.e., reducing the effectiveness of pre-planning processes). However, in line with CPH, it was hypothesised that late in learning the introduction of pressure would lead to increased spatial variability at later kinematic markers due to the interruption of proceduralised and reflexive online control processes (Lawrence et al., 2012b).

## Method

### Participants

Twenty-four right-handed adults (13 female, 11 male) aged 19–40 years (*M* = 25.3, SD ± 5.5) volunteered to partake in the study. Participants were randomly assigned to either a pressure group or control group. Random assignment was stratified by gender (pressure group 6 female, 6 male; control group 7 female, 5 male). All participants had no prior experience in the experimental task and were naive to the hypotheses being tested. Written informed consent was gained from all participants and the experiment was conducted in accordance with the Institutions Ethics for research involving human participants.

### Apparatus

The aiming movements were performed with a stylus on a Calcomp III digitising tablet (size = 122 × 91.5 cm, sample rate = 200 Hz) positioned horizontally in front of participants. Movements were performed with the right hand in a left to right direction along a track-way. The track-way constrained movement to ensure the task had no directional requirement. The position of the stylus was illustrated by a white cursor consisting of a vertical line (2 cm in length and 0.2 cm in width) on a 37 in. Mitsubishi Diamond Pro monitor (refresh rate = 85 Hz) located 33 cm in front of the participants and 20 cm above the tablet. There was one to one mapping between the movement of the stylus and the movement of the cursor. A home position and target were presented on the monitor 12 cm to the left and right of the participants’ midline, respectively. The home position and target were identical in dimensions to that of the cursor with the exception that the home position was green in colour and the target was red. The participants arm and hand were obscured by an opaque shield at all times.

### Procedure

At the beginning of the experiment, the home, target and cursor representing the position of the pen appeared on the monitor and remained visible throughout the experiment. Participants were required to place the cursor on the home position and then fixate on the target. A warning tone was then presented. This was followed by a variable fore period (1500–2500 ms) before a final tone was presented to signal the start of the trial. Participants were then required to move the cursor from the home position and come to a complete stop as close to the target as possible. Participants were instructed that reaction time was not important but that the movement must be completed within a 400 ms criterion movement time. This criterion movement time was selected as it allows sufficient processing time for both online and offline correction of movement errors (Khan et al., 2003a, 2003b). Participants were also told that they should make the movement as smooth as possible.

Each participant observed five demonstration trials of the appropriate movement and then completed five practice trials. Following this, participants performed a total of 420 trials over a 2 day period, with trials grouped into 14 blocks of 30 trials. Numerical feedback for movement time (ms) and a point score^1^ were presented on the monitor after each trial. The pressure group were transferred to a pressure condition for block 2 (i.e., early in practice) and block 14 (i.e., late in practice). The control group performed under normal conditions for all blocks of trials (i.e., without any pressure manipulation for block 2 or block 14).

The pressure manipulation consisted of a combination of socially evaluative instructions and monetary incentives, both of which have been shown to effectively invoke self-reported anxiety in laboratory settings (e.g. Hardy, Mullen, & Jones, 1996; Lawrence et al., 2012b; Mullen & Hardy, 2000; Wilson et al., 2007). Specifically, at the beginning of both the early and late anxiety transfers, participants were informed that they would be entering a competition block where the individual who performed best at the task would win £50. However, the participant was also informed that they were to be paired with a partner. They were informed that both they and their partner had to improve their performance by 20 % in comparison to their previous 30 trials to be eligible for the monetary prize. Furthermore, if successful, their individual names would be placed on the leader board for other participants to view. However, if either participant did not improve by 20 %, both team-members would not be eligible to enter the leader board and would forfeit the possibility of winning the monetary prize. Participants were then informed that the partner they had been randomly paired with had already completed the task and had improved by the criterion 20 % and were therefore reliant on their partner increasing performance by the required 20 % if both parties were to be eligible to win the prize.^2^ Furthermore, participants were also told that their performance was being video recorded and would be subsequently analysed by members of staff and PhD students (e.g. Mullen & Hardy, 2000; Cooke, Kavussanu, McIntyre, & Ring, 2010). The actual sole determinant of monetary reward was the participant who had the highest performance increase above the criterion 20 % (i.e., in line with the experimental instructions provid ed, the participant who increased performance by the required amount *and* performed the best out of all the participants won the £50). All other manipulations were part of the ethically approved pressure deception. To monitor and ensure that cognitive anxiety was successfully invoked by the pressure manipulation, all participants completed the Mental Readiness Form-3 (MRF-3; Krane, 1994) (see below for specific details) on four separate occasions; at the start of acquisition; the start of early transfer; the start of the last block of acquisition; and the start of late transfer. Mental effort was also monitored by completing the Rating Scale for Mental Effort (RSME; Zijlstra, 1993) (see below for specific details) on completion of each of these four experimental phases.^3^

### Psychological measures

#### Cognitive state anxiety

Cognitive state anxiety was measured using the Mental Readiness Form-3 (MRF-3; Krane, 1994). The MRF-3 has three bipolar 11-point likert scales that are anchored at the extremes with *not worried* and *worried* for cognitive anxiety; *not tense* and *tense* for somatic anxiety; and *confident and not confident* for self-confidence. For the purpose of this study only the cognitive anxiety scale was used. This measure is a shorter alternative to the Competitive State Anxiety Inventory-2 (CSAI-2; Martens, Burton, Vealey, Bump, & Smith, 1990) but retains correlation coefficients with the CSAI-2 of 0.76 for cognitive anxiety, 0.69 for somatic anxiety and 0.68 for self-confidence (Krane, 1994).

#### Mental effort

Mental effort was measured using the Rating Scale for Mental Effort (RSME; Zijlstra, 1993). The scale consists of a vertical axis with numbers ranging from 0 to 150, with nine category anchors, including at the extremes; 3 (*No Mental Effort at All*) and 114 (*Extreme Mental Effort*). This measure strongly correlates with psychophysiological measures of mental effort such as heart rate variability and event related potentials (Veltman & Gaillard, 1996; Zijlstra, 1993).

### Kinematic measures

#### Data reduction and dependent variables

The displacement data for each trial were filtered using a second-order dual-pass Butterworth filter with a low-pass cutoff frequency of 10 Hz. Instantaneous velocity data were obtained by differentiating the displacement data using a two-point central finite difference algorithm. This process was then repeated on the velocity data to obtain acceleration data. To locate the beginning of the movement, peak velocity was first obtained. The velocity profile was then traversed backwards in time until the velocity fell below 1 mm/s. The end of the movement was defined as the first point in time following peak velocity in which the absolute velocity of the stylus fell below 1 mm/s. This criteria for the end of the movement meant that trajectories could not contain a reversal in direction. These analyses allowed the production of four kinematic markers for each trial; peak acceleration (p*k*a), peak velocity (p*k*v), peak negative acceleration (p*k*na) and movement end (end). This procedure was completed in *real time* through a process of raw data being passed from the task software (Visual Basic) to the custom written Labview analysis programme. The Labview programme then also fed back information regarding MT and point score to Visual Basic so that feedback regarding these measures could be displayed to participants on the monitor screen after each trial. This entire sequence took approximately 400 ms.

Performance measures included movement time, absolute error and variable error (i.e., within-participant standard deviation of directional error) at the end of movement. Error was calculated from the centre of the movement cursor to the centre of the target marker. To enable the investigation of spatial variability throughout the movement, the within-participant standard deviation in the distance travelled at each kinematic landmark (i.e. p*k*a, p*k*v, p*k*na and end) was calculated (see Khan et al., 2006 for a review).

### Data analysis

To analyse the effect of pressure on the psychological measures of cognitive anxiety and mental effort, separate 2 group (pressure; control) × 4 block (acquisition block 1; early transfer; acquisition block 12; late transfer) ANOVAs with repeated measures on the second factor were performed. To analyse the effect of block (experimental phase) on Points Score, MT, AE, and VE, separate 2 group (pressure; control) × 14 block (acquisition block 1; early transfer; acquisition block 2; acquisition block 3; acquisition block 4; … acquisition block 11; acquisition block 12; late transfer) ANOVAs with repeated measures on the second factor were conducted. Finally, to analyse the effect of pressure on spatial variability throughout the movement as a function of skill level, a 2 group (pressure, control) × 4 experimental phase (acquisition block 1; early transfer; acquisition block 12; late transfer) × 4 kinematic marker (p*k*a, p*k*v, p*k*na, end) ANOVA with repeated measures on the last two factors was conducted. For all analyses, Greenhouse–Geisser adjustments were made when sphericity was violated and, unless otherwise stated, Post-hoc tests were performed using Tukey HSD methods (*p* < 0.05).

## Results

### Psychological measures

#### Cognitive state anxiety

**Fig. 1:**
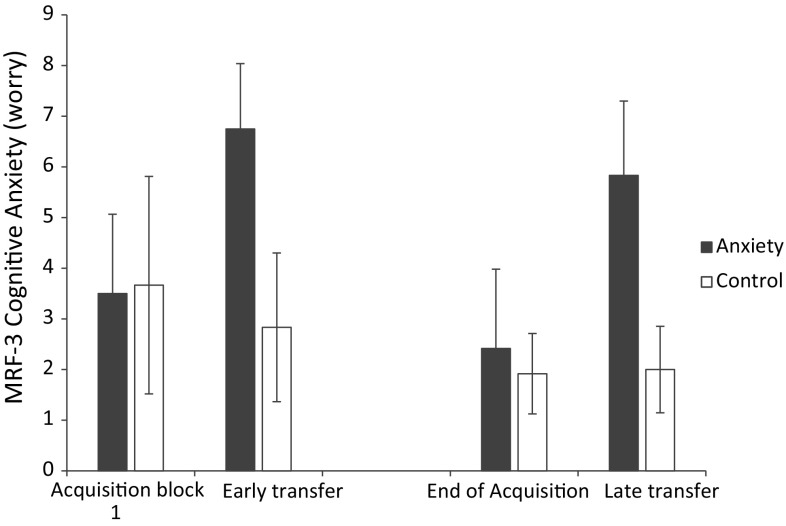
Mean (±SEm) cognitive anxiety for the control and anxiety groups at the first block of acquisition, early transfer, the last block of acquisition, and late transfer

The analysis of variance revealed significant main effects for group (*F*_(1,22)_ = 18.84, *p* < 0.001, $\eta_{\text{p}}^{ 2}$ = 0.46) and block (*F*_(3,66)_ = 26.73, *p* < 0.001, $\eta_{\text{p}}^{ 2}$ = 0.55), together with a significant group × block interaction (*F*_(3,66)_ = 26.13, *p* < 0.001, $\eta_{\text{p}}^{ 2}$ = 0.54). Breakdown of the interaction revealed that whilst cognitive state anxiety remained constant for the control group it significantly increased in the pressure group after both the early and late transfer pressure manipulations (see Fig. 1).

#### Effort

**Fig. 2:**
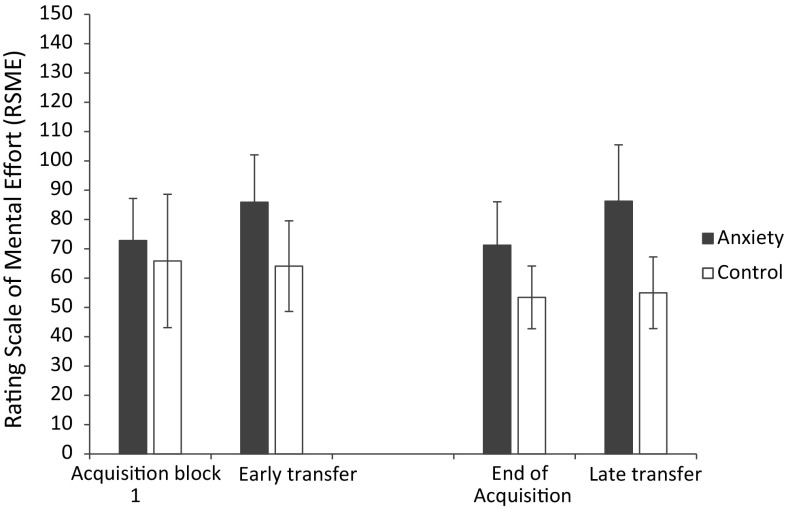
Mean (±SEm) mental effort for the control and anxiety groups at the first block of acquisition, early transfer, the last block of acquisition, and late transfer

As shown in Fig. 2, effort data analysis revealed significant main effects for group (*F*_(1,22)_ = 14.92, *p* = 0.001, $\eta_{\text{p}}^{ 2}$ = 0.40) and block (*F*_(3,66)_ = 4.64, *p* = 0.005, $\eta_{\text{p}}^{ 2}$ = 0.17), together with a significant group × block interaction (*F*_(3,66)_ = 4.24, *p* = 0.008, $\eta_{\text{p}}^{ 2}$ = 0.16). Breakdown of the interaction revealed that the mental effort of the control group remained constant whereas the mental effort of the pressure group significantly increased in both early and late transfer pressure manipulations.

### Movement time

**Fig. 3:**
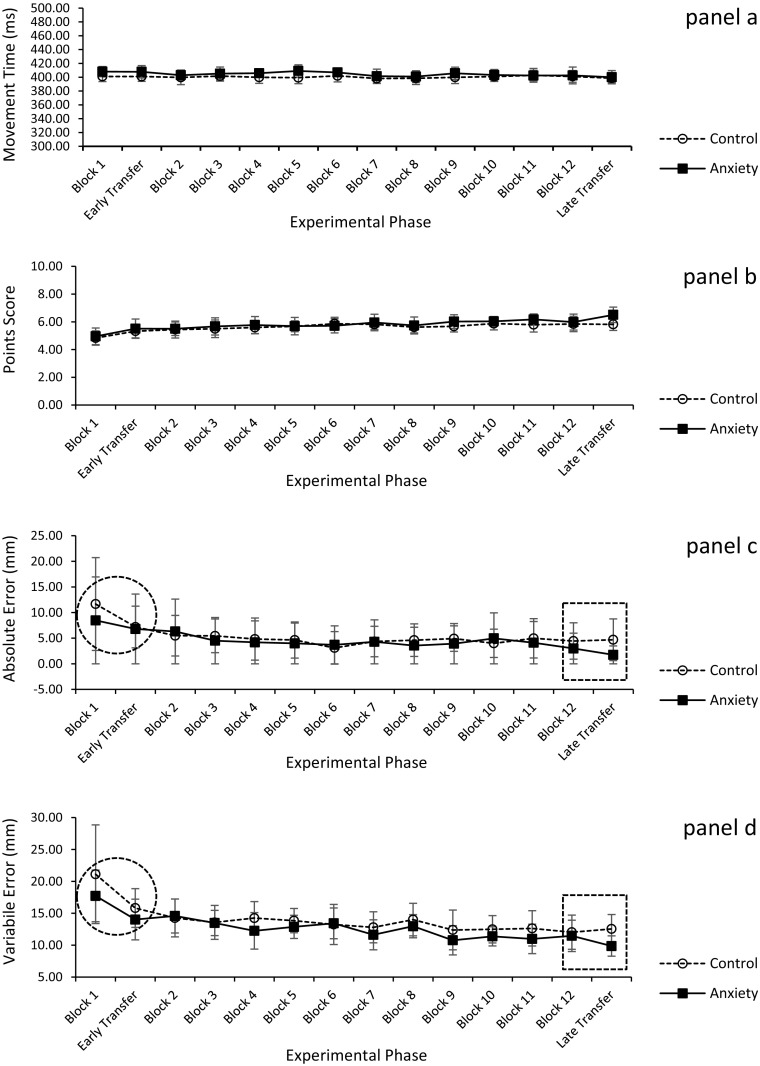
Mean (±SEm) MT (**a**), points score (**b**), absolute error (**c**), and variable error (**d**) as a function of experimental phase. The *dashed rectangle* on **c** and **d** indicate the observation of a significant (*p* < 0.05) group × experimental phase interaction and the *dashed circle* indicate the observation of a significant (*p* < 0.05) main effect of experimental phase

As shown in Fig. 3a, analysis of movement time revealed non-significant main effects for group (*F*_(1,22)_ = 3.94, *p* = 0.06, $\eta_{\text{p}}^{ 2}$ = 0.15) and block (*F*_(13,286)_ = 1.57, *p* = 0.09, $\eta_{\text{p}}^{ 2}$ = 0.07), and a non-significant group × block interaction (*F*_(13,286)_ = 0.89, *p* = 0.57, $\eta_{\text{p}}^{ 2}$ = 0.04).

### Points score

As shown in Fig. 3b, the analysis of the points score data revealed a significant main effect for block (*F*_(13,286)_ = 11.70, *p* < 0.001, $\eta_{\text{p}}^{ 2}$ = 0.35) with points increasing over the course of acquisition (block 2 to block 12). In reference to the study’s planned experimental phases of interest (i.e., early and late transfer), the main effect of block also revealed that the point score of both groups significantly increased from the first block of acquisition to early transfer and remained constant between the last block of acquisition and late transfer. The main effect for group (*F*_(1,22)_ = 2.20, *p* = 0.15, $\eta_{\text{p}}^{ 2}$ = 0.09) and the group × block interaction (*F*_(13,286)_ = 1.13, *p* = 0.38, $\eta_{\text{p}}^{ 2}$ = 0.05) were non-significant.

### Absolute error and variable error

The separate analyses of the AE and VE data over all 14 trial blocks revealed only significant main effects for block (*F*_(1,22)_ = 5.69, *p* = 0.026, $\eta_{\text{p}}^{ 2}$ = 0.21 and *F*_*(*1,22)_ = 15.97, *p* = 0.001, $\eta_{\text{p}}^{ 2}$ = 0.42, respectively) with both error values decreasing from block 2 to block 12 (see Fig. 3c, d, respectively).

Because our hypotheses centred on predictions associated with the introduction of pressure early and late in learning, we performed planned comparisons at these time points as they are preferable to the omnibus significance test because they allow evaluation of the effects at their theoretical importance. That is, with the omnibus test model, one can only strictly compare pairs of groups at a specific theorised repeated measure if the first stage of the ANOVA method shows an overall statistically significant effect across all of the repeated measures. Since our predictions were based on planned comparisons at early and late transfer, we isolated the effects of pressure on AE and VE early in learning by conducting separate 2 group (pressure; control) × 2 block (acquisition block 1; early transfer) ANOVAs with repeated measures on the second factor. Similarly, to isolate the effects of pressure on AE and VE late in learning, we conducted identical analyses [2 group (pressure; control) × 2 block (acquisition block 12; late transfer) ANOVAs with repeated measures on the second] on the late transfer data.

Results at early transfer revealed only significant main effects for block (AE; *F*_(1,22)_ = 5.69, *p* = 0.026, $\eta_{\text{p}}^{ 2}$ = 0.21 and VE; *F*_*(*1,22)_ = 15.97, *p* = 0.001, $\eta_{\text{p}}^{ 2}$ = 0.42, respectively). Further examination of the means indicated that both AE and VE significantly decreased between block 1 (AE = 10.07 mm; VE = 19.35 mm) and early transfer (AE = 6.99 mm; VE = 14.85 mm) (see Fig. 3c, d). The main effects for group (AE; *F*_(1,22)_ = 0.57, *p* = 0.45, $\eta_{\text{p}}^{ 2}$ = 0.03; VE; *F*_(1,22)_ = 2.65, *p* = 0.12, $\eta_{\text{p}}^{ 2}$ = 0.10) and the group × block interactions (AE; *F*_(1,22)_ = 1.18, *p* = 0.29, $\eta_{\text{p}}^{ 2}$ = 0.05; VE; *F*_(1,22)_ = 0.48, *p* = 0.49, $\eta_{\text{p}}^{ 2}$ = 0.02) were non-significant. At late transfer, the analyses of both AE and VE revealed significant main effects for group (AE; *F*_(1,22)_ = 4.14, *p* = 0.050, $\eta_{\text{p}}^{ 2}$ = 0.158; VE; *F*_(1,22)_ = 4.30, *p* = 0.05, $\eta_{\text{p}}^{ 2}$ = 0.163), non-significant main effects for block (AE; *F*_(1,22)_ = 2.43, *p* = 0.134, $\eta_{\text{p}}^{ 2}$ 0.099; VE; *F*_(1,22)_ = 4.13, *p* = , 0.234, $\eta_{\text{p}}^{ 2}$ = 0.064), and a significant group × block interactions (AE; *F*_(1,22)_ = 4.97, *p* = 0.036, $\eta_{\text{p}}^{ 2}$ = 0.184; VE; *F*_(1,22)_ = 4.80, *p* = 0.04, $\eta_{\text{p}}^{ 2}$ = 0.179). Breakdowns of these interactions revealed that whilst the performance of both AE and VE remained constant between the last block of acquisition and late transfer for the control group it significantly improved for both these measures in the pressure group (see Fig. 3c, d, respectively).

### Spatial variability

**Fig. 4:**
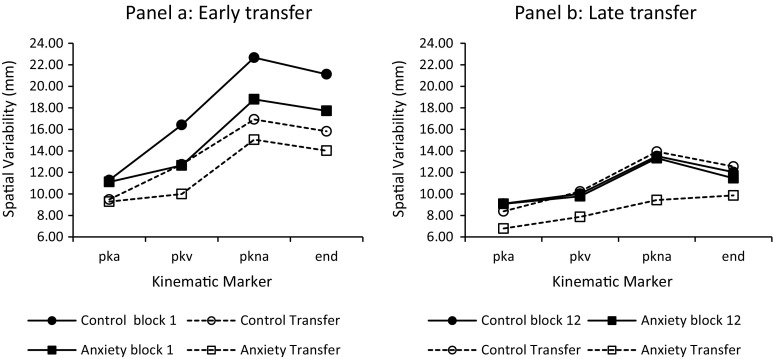
Mean spatial variability as a function of kinematic marker (p*k*a = peak acceleration; p*k*v = peak velocity; p*k*na = peak negative acceleration; end = movement end) for the effects of early transfer (**a**) and late transfer (**b**)

As shown in Fig. 4, the omnibus analysis of spatial variability revealed significant main effects for block (*F*_(3,66)_ = 31.11, *p* < 0.001, $\eta_{\text{p}}^{ 2}$ = 0.57) and kinematic marker (*F*_(3,66)_ = 54.41, *p* < 0.001, $\eta_{\text{p}}^{ 2}$ = 0.71). Specifically, variability significantly increased as the movement unfolded from peak acceleration to peak negative acceleration and overall variability significantly decreased from the acquisition block 1 and early transfer experimental phases to the acquisition block 12 and late transfer experimental phases. Of more significant interest was the observation of block × kinematic marker (*F*_(9,198)_ = 10.20, *p* < 0.001, $\eta_{\text{p}}^{ 2}$ = 0.31) and group × block × kinematic marker interactions (*F*_(9,198)_ = 2.01, *p* = 0.04, $\eta_{\text{p}}^{ 2}$ = 0.10). Similar to the planned comparisons for the AE and VE, we investigated the two-way interaction by analysing spatial variability throughout the limb trajectory separately at the repeated measures time points of hypothesised importance. That is, to investigate the effects of pressure early in learning we conducted a 2 group (pressure versus control) × 2 block (acquisition block 1 versus early transfer) × 4 kinematic marker (p*k*a, p*k*v, p*k*na, end) mixed model ANOVA with repeated measures on the last two factors. Similarly, we conducted a separate 2 group (pressure versus control) × 2 block (the last block of acquisition versus late transfer) × 4 kinematic marker (p*k*a, p*k*v, p*k*na, end) mixed model ANOVA with repeated measures on the last two factors to investigate the effects late in learning. The analysis at early transfer revealed a significant main effect for block (*F*_(1,22)_ = 17.11, *p* < 0.001, $\eta_{\text{p}}^{ 2}$ = 0.44), a significant main effect for kinematic marker (*F*_(1.49, 32.82)_ = 47.63, *p* < 0.001, $\eta_{\text{p}}^{ 2}$ = 0.68), and a significant block × kinematic marker interaction (*F*_(3, 66)_ = 6.57, *p* < 0.001, $\eta_{\text{p}}^{ 2}$ = 0.23). Breakdown of the interaction revealed that variability significantly increased from peak acceleration to peak negative acceleration and then levelled off (was not significantly different) between peak negative acceleration to movement end for both experimental phases. However, the increase in variability between peak acceleration and peak negative acceleration was significantly greater in block 1 compared to early transfer (see Fig. 4a).

To further assess whether the form of the variability profiles differed between acquisition block 1 and early transfer, the ratios in spatial variability between these two experimental phases were calculated for each kinematic marker (see Khan et al., 2006). These data were submitted to separate (Bonferroni adjusted for multiple tests) pairwise comparisons. Analysis revealed non-significant differences between the ratio’s at each kinematic marker demonstrating that the form of the variability profiles did not significantly differ (p*k*a = 0.92; p*k*v = 0.84; p*k*na = 0.84; end = 0.79; mean difference (p*k*a − p*k*v) = 0.08, *p* = 1.00; mean difference (p*k*a − p*k*na) = 0.08, *p* = 1.00; mean difference (p*k*a − end) = 0.14, *p* = 0.78; mean difference (p*k*v − p*k*na) = 0.08, *p* = 1.00; mean difference (p*k*na − end) = 0.08, *p* = 1.00).

The spatial variability data for the planned comparisons late in learning are shown in Fig. 4b. The analysis revealed significant main effects for block (*F*_(1,66)_ = 8.74, *p* < 0.05, $\eta_{\text{p}}^{ 2}$ = 0.28) and kinematic marker (*F*_(1.66,36.44)_ = 36.10, *p* < 0.001, $\eta_{\text{p}}^{ 2}$ = 0.62), together with a significant group × block interaction (*F*_(1, 22)_ = 10.47, *p* < 0.05, $\eta_{\text{p}}^{ 2}$ = 0.32). Breakdown of the interaction revealed that variability was significantly lower at late transfer compared to the last block of acquisition for the pressure group (last block of acquisition mean = 10.91, late transfer mean = 8.48). In contrast there was no significant difference in variability for the control group (last block of acquisition mean = 11.16, late transfer mean = 11.27). All other interactions were non-significant (*p* > 0.05).

A supplementary 2 block (acquisition block 12; transfer) × 4 kinematic marker (p*k*a; p*k*v; p*k*na; end) doubly repeated measures follow-up test was performed on the pressure group data to examine which kinematic markers were responsible for the observed reduction in variability. The analysis revealed significant main effects for block (*F*_(1,11)_ = 39.46, *p* < 0.001, $\eta_{\text{p}}^{ 2}$ = 0.78) and kinematic marker (*F*_(1.51,16.52)_ = 12.09, *p* < 0.001, $\eta_{\text{p}}^{ 2}$ = 0.52), together with a significant block × kinematic marker interaction (*F*_(3, 33)_ = 3.45, *p* < 0.05, $\eta_{\text{p}}^{ 2}$ = 0.24). Breakdown of the interaction revealed that in the last block of acquisition (e.g., the low pressure condition), variability significantly increased from peak acceleration to peak negative acceleration and then significantly decreased from peak negative acceleration to movement end. However, in late transfer (e.g., high pressure condition) variability significantly increased from peak acceleration to peak negative acceleration and then remained constant between peak negative acceleration and movement end. In addition, variability was significantly lower in late transfer compared to the last block of acquisition at all kinematic markers.

To further assess whether the form of the pressure group variability profiles differed between the low (last block of acquisition) and high (late transfer) pressure conditions, the ratio in spatial variability between the low and high pressure conditions was calculated for each participant (see Khan et al., 2006). These data were submitted to separate (Bonferroni adjusted for multiple tests) pairwise comparisons. These analyses revealed that the ratio of the variability profiles remained constant from peak acceleration to peak negative acceleration (p*k*a = 1.41; p*k*v = 1.30; p*k*na = 1.42; mean difference (p*k*a − p*k*v) = 0.11, *p* = 1.00; mean difference (p*k*a − p*k*na) = −0.01, *p* = 1.00; mean difference (p*k*v − p*k*na) = 0.24, *p* = 1.00), but then significantly decreased between peak negative acceleration and movement end (1.17); mean difference (p*k*na − end) = −0.249, *p* = 0.006. Thus, the form of the variability profiles were significantly different for the pressure group between the last block of acquisition and late transfer.

## Discussion

### Psychological measures and summary

Previous research has shown that pressure can influence the performance of sensorimotor skills. However, the effects of pressure on the processes that support performance are far from clear. The aim of the present study was to concurrently examine the effect of pressure on both the preplanning and online control phases of movement execution at both the early and late phases of learning. Self-report data from the MRF-3 indicated that cognitive state anxiety was successfully invoked by the experimental pressure manipulation. Levels of state anxiety in both the pressure transfer conditions were similar to previous laboratory pressure manipulations (e.g. Vine & Wilson, 2011; Wilson et al., 2007; Wilson, Wood, & Vine, 2009) and the increased state anxiety that occurred under pressure manipulations was coupled with a significant increase in mental effort. In addition, analysis of endpoint error revealed that performance increased at late pressure transfer. However, analysis of kinematic variability throughout the movement indicated that this increase in performance was due to participants adopting strategies to improve movement planning in response to pressure reducing the effectiveness of the online control system.

### Performance measures

#### Early transfer

We had hypothesised that the pressure group would outperform the control group at early transfer due to self-focus theories indicating that novice performance should benefit from attention being placed on the step-by-step execution of skill (e.g. Beilock, Carr, MacMahon, & Starkes, 2002; Gray, 2004). However, when transferring participants to pressure conditions early in learning, accuracy results showed an absence of any group differences in endpoint absolute error. Instead, the results showed a comparable improvement in performance from the first block of acquisition to early transfer for both the control and pressure group. By using Khan & colleagues variability methodology (Khan et al., 2003a, 2003b) we were able to examine whether these changes in performance were due to pre-planning or online control. Specifically, this methodology involved the calculation and analysis of the within-subject standard deviation of distance travelled for peak acceleration, peak velocity, peak negative acceleration, and movement end. In support of previous research (e.g. Khan et al., 2003b), the analyses of acquisition block 1 (the first 30 trials) revealed that variability increased from the start of the movement until peak negative acceleration, before then decreasing between peak negative acceleration and movement end. This variability profile indicates that afferent information was utilised online to regulate movement during execution (see Khan et al., 2006 for a review). Importantly, the form of the variability profile did not change for either the control or the pressure group between the first block of acquisition and early transfer. Specifically, the analysis of the ratios between the two experimental phases revealed no significant differences. However, the variability at early transfer was significantly lower at each kinematic marker compared to the acquisition block 1. Researchers have suggested that movement planning processes are reflected in changes or reductions in variability to kinematic markers up to and including peak velocity (Lawrence et al., 2006, 2011). Thus, the reduction in variability at peak velocity in early transfer suggests that all participants began to plan movement parameters more accurately after an initial 30 trials of practice in the current novel target directed aiming task. Given that these planning processes increased in both the control and the pressure group, it is unlikely that they were specific to the introduction of pressure. Rather, the observed increase in planning may simply be a reflection of the processes involved in early learning and motor programme development.

#### Late transfer

Results at late transfer revealed that absolute error decreased only for the pressure group. This finding was somewhat contrary to our hypothesis, as we expected that performance would be detrimentally affected by pressure at later stages of learning. However, whilst unexpected, previous research has revealed that expert performers can increase task accuracy when under conditions of perceived pressure through increased mental effort (Cooke et al., 2010). The results of the effort data are in line with this proposal since mental effort increased from the last block of acquisition to late transfer in the pressure group.

The variability profiles of the control group for both acquisition and transfer did not differ and significantly increased from the start of movement until peak negative acceleration before significantly decreasing from peak negative acceleration to movement end. This variability profile is indicative of online control processes being utilised towards the latter stages of movement trajectories to home in on the target by continually updating limb and target location and reducing the discrepancy between the two (Elliott et al., 2010; Khan et al., 2006). For the pressure group, the variability profiles between acquisition and transfer were significantly different. Specifically, whilst variability in acquisition was similar to the control group and indicative of online control (i.e., variability significantly increased from the start of movement until peak negative acceleration before significantly decreasing from peak negative acceleration to movement end), the variability profile at transfer significantly increased up until to peak negative acceleration and then remained constant between peak negative acceleration and movement end. The analysis of the ratios between the last block of acquisition and transfer confirmed that the form of the variability profiles for the pressure group were different between acquisition and transfer. Specifically, the analysis revealed that the ratio of the variability profiles remained constant from peak acceleration to peak negative acceleration, but then significantly decreased between peak negative acceleration and movement end, indicating a reduction in online control processes in transfer (i.e., under pressure).

### Theoretical explanations and implications

#### Self-focus

As hypothesised, the reduction in online control processes following the introduction of pressure late in learning offers support for the conscious processing hypothesis (Masters, 1992). Conscious processing hypothesis posits that pressure to perform and the ensuing anxiety negatively affects performance through breakdowns of automaticity, as a result of efforts to control the mechanics of the movement during the motor output (Maxwell & Masters, 2004). Using a similar methodology to the present study, Lawrence et al. (2012b) found evidence to support this prediction when participants were transferred to conditions of pressure after only 90 trials. Thus, because online process occur during movement and are said to be reflexive and lie outside of working memory (Briere & Proteau, 2011; Proteau et al., 2009; Veyrat-Masson et al., 2010; Lawrence et al., 2012b), we propose that the presence of pressure in the current experiment led to conscious attention to these automatic and attention-free online control processes. This resulted in an increase in skill focused attention and subsequent reinvestment, leading to a breakdown of the normally automatic online control processes; reflected in an increase in variability at the latter kinematic landmarks. These findings extend those of Lawrence et al. (2012b) by indicating that late in learning the use of online control processes to ensure movement accuracy during control conditions are reduced and less effective following the introduction of pressure.

#### Distraction

Whilst the reduction of online control processes under late pressure transfer offers support for the conscious processing hypothesis of Masters (1992), the data are not entirely dismissive of pressure–performance interactions associated with the processes proposed within Eysenck et al’s (2007) attentional control theory. Specifically, participants in the pressure group adjusted the planning of movement parameters (increased the accuracy of their pre-planning processes) between acquisition and transfer. Support for this was observed in the pressure group in the form of a reduction in variability as early as peak acceleration in the late transfer compared to last block of acquisition. Indeed, Lawrence et al. (2006, 2011) propose that increases in planning processes manifest themselves in a reduction in early kinematic markers, namely peak acceleration and peak velocity. Furthermore, effective pre-planned parameterisation of an appropriate response is achieved via relatively effortful and non automatic processes (Beilock, Jellison, Rydell, McConeell, & Carr, 2006; Schmidt, Zelaznik, Hawkinsm Frank, & Quinn, 1979), is proposed to involve a degree of conscious control (Klatzky et al., 1987, 1999), and is therefore open to the influence of cognitive factors (Glover & Dixon, 2002; Glover et al., 2004). Attentional control theory may therefore be able to explain the observed improvements in pre-planning within the current experiment. Whereby pre-planning performance effectiveness improved under pressure transfer through the release of additional self-evoked resources (e.g., effort). This improved performance effectiveness was achieved at the expense of performance efficiency, as the additional effort was released as a strategy to compensate for the working memory space occupied by the state anxiety that arose because of increased pressure to perform. Therefore, for the current study, the observed decrease in variability at kinematic markers associated with pre-planning indicates that the parameterisation of movement may have benefited from the release of anxiety-induced self-evoked resources; in this instance, additional effort. As both state anxiety and mental effort increased under the pressure manipulation, we suggest that this improvement in pre-planning effectiveness was achieved despite degraded planning efficiency. We propose that participants adopted this strategy of increasing effort, and thus the accuracy of the cognitive control processes associated with pre-planning, in an attempt to reduce the performance decrements associated with a reduction in the use of online control processes under pressure induced anxiety (Lawrence et al., 2012b).

Recently, Englert and Bertrams (2012, 2013, 2015) have observed and proposed that the release of self-evoked resources to control the effects of state anxiety on performance is dependent on one’s self control strength. Specifically, the volitional inhibition of attentional shifts from goal-orientated to stimulus-driven processing to maintain performance under conditions of pressure, depends on the momentary availability of self-control strength regarding these resources. That is, because all acts of self-control are proposed to be analogous to that of a muscle (Schmeichel & Baumeister, 2010), the resources associated with these acts are limited. Therefore, the resources available for self-regulatory processes to control performance under situations of heighted pressure to perform can become depleted and ineffective if not replenished (e.g., if one is in a state of ego depletion, see Baumeister, Bratslavsky, Muraven, & Tice, 1998). In these situations, an individual should demonstrate the choking phenomenon under pressure conditions because they cannot invest the required amount of self-regulatory processes to inhibit the shift in attention from goal-orientated to stimulus driven task processing. In the current study, it appears that when pressure was manipulated, both self-reported anxiety increased *and* participants were able to release additional self-evoked resources (e.g., mental effort) in an attempt to control performance. Therefore, in line with Englert and Bertrams (2012, 2013, 2015), participants were able to alleviate the effects of pressure on performance because their self-control strength was sufficient enough to allow the release of self-evoked resources. These self-evoked resources (i.e., mental effort in the current study) permitted participants to adapt their movement control strategies from a predominantly online to offline control strategy when producing target directed aiming movements. Because this performance strategy was adopted under the pressure manipulation, *and* following the release of additional mental effort, one can propose that participants in the experimental group had sufficient self control strength to permit the self-evoked resources necessary to maintain performance under pressure. It would be interesting to explore this *pressure–performance and self*-*evoked resource*-*self strength* interaction further within the context of changes to online versus offline movement control strategy. To achieve this, future research could adopt experimental protocols similar to that of Bertrams, Englert, Dickhauser, and Beaumeister (2013) by investigating the changes to performance and motor control strategies following the introduction of pressure between participants who are either in a state of ego depletion or not.

#### Self focus versus distraction

Initially, the performance data of the current experiment point to a CPH or reinvestment theory (Masters, 1992) of explanation for the pressure–performance relationship observed. That is, it was the reflexive and non-conscious processes of online limb adjustment (proposed to lie outside of working memory; Briere & Proteau, 2011; Proteau et al., 2009; Veyrat-Masson et al., 2010; Lawrence et al., 2012b) that suffered performance decrements following the introduction of pressure. Thus, one could conclude that the presence of pressure led to conscious attention to the automatic and attention-free online control processes, resulting in increased skill focused attention, subsequent reinvestment, and ultimately a breakdown of the normally automatic online control processes; or put simply, reinvestment occurred under conditions of pressure. However, the reduction in the effectiveness of automatic online control processes was accompanied by increases in self-evoked resources (i.e., mental effort) *and* increases in the effectiveness of the relatively effortful and non-automatic processes (Beilock et al., 2006; Schmidt et al., 1979) associated with offline control (i.e., the effective pre-planned parameterisation of appropriate responses). Because the parameterisation of movement appears to have benefited from the release of state anxiety-induced self-evoked resources; in this instance, additional effort, we propose that the improvement in pre-planning effectiveness was achieved via the processing efficiency aspect of Esyneck et al’s ACT (2007). Furthermore, we propose that participants adopted a strategy of increasing self-evoked resources because (a), in line with Englert and Bertram (2015), they had sufficient self control strength to do so, and (b) this increased self-evoked release of resources led to an increase in the attention demanding pre-planning processes. Not because these processes are those more likely to be associated with the goal-orientated attentional control as proposed in ACT, but rather because this strategy helped to maintain performance in response to a decrement in the effectiveness of one’s automatic online control processes (i.e., CPH or reinvestment). Therefore, we conclude that the pressure–performance data are supportive of the performance maintaining proposals within Eysenck et al’s (2007) ACT and Englert and Bertrams (2015) integration of ACT and the strength model of self control in response to changes in the control of automatic online movement control processes because of Masters (1992) CPH and reinvestment proposals. That is, participants adopted movement control strategies that involved the release of self-evoked resources to increase the effortful and conscious processes associated with pre-planning/offline control to maintain performance in the face of a reinvestment based reduction in the effectiveness of the automatic and non conscious online processes (i.e., pressure affected performance based on a combination of CPH and ACT).

#### Strategic optimisation

Research explicitly investigating the strategic optimisation of pre-planning and online trajectory adjustments has revealed that individuals attempt to plan movements that reduce the likelihood of the need for time consuming and energy intensive online adjustments (Khan, Elliott, Coull, Chua, & Lyons, 2002; Khan & Franks, 2000; Lyons, Hansen, Hurding, & Elliott, 2006; Meyer, Abrams, Kornblum, Wright, & Smith, 1988; Oliveria, Elliott, & Goodman, 2005). For example, Meyer et al.’s (1988) optimized submovement model proposes that a balance is made between movement velocity and greater endpoint error when planning actions. That is, participants strategically plan movements to reach an optimisation between the speed of movement and any associated online corrective adjustments to ensure targets are reached as quickly, accurately, and efficiently as possible in any given confine. In addition, recent research has revealed that participants adopt strategies of pre-planning target directed aiming movements made against gravity (i.e., in the vertical direction) to avoid online corrective adjustments (Bennet, Elliott, & Rodacki, 2012; Elliott et al., 2014; Lyons et al., 2006). When moving downwards (with gravity) to targets, compared to upwards (against gravity), Elliott et al. (2014) have observed that movements are often planned to land only in the vicinity of the target region without engaging in potentially inefficient online movement adjustments. Furthermore, any online adjustments that do occur are shorter in duration and distance in the downward compared to upward aiming directions; presumably to prevent overshooting a downward target that would then require a costly reversal in direction and corrective adjustment against (rather than with) gravity. These research findings suggest that participant’s pre-planning is consciously designed to both reduce the need for online adjustments and optimise movements in relation to the time and energy expenditures available within the environmental context (Elliott et al., 2010; Meyer et al., 1998). In the current experiment, we are proposing that the change in pre-planning and online adjustments between the last block of acquisition and the pressure transfer was a result of strategic optimisation (following the release of self-evoked resources) to meet the environmental context. Both the data of Lawrence et al. (2012b) and that of late transfer in the current investigation revealed that the effectiveness of online adjustments is significantly reduced under pressure conditions compared to normal (low pressure) control conditions. Therefore, it is possible that participants adopted movement strategies that increased the pre-planning accuracy of limb trajectories under pressure conditions to avoid the need for inefficient and costly online adjustments. The experimental design, data acquisition, and data reduction procedures used in the current study were designed to reduce the parsing of initial movement impulses and subsequent discrete submovements described in Meyer et al.’s (1988), optimized submovement model in favour of analysing more continuous online adjustments (see Khan et al., 2006). It is recommended that future research adopt data acquisition designs that explicit decouple initial impulses and discrete online adjustments to further investigate our claim that under pressure conditions participants increase the accuracy of their initial (pre-planned) impulses to reduce the requirement for costly and inefficient online corrective adjustments.

### Applied implications

Based on the findings of the current experiment and those of Lawrence et al. (2012b), we suggest that interventions aiming to aid expert performance in pressure conditions should focus on improving movement preparation, while avoiding lapses into controlling the production of the movement. Indeed, it is possible that interventions that have previously been shown to be effective may do so by aiding pre-planning processes. For example, Mesagno and Mullane-Grant (2010) showed that merely having a temporally consistent preparation phase before taking Australian football kicks offered similar performance benefits when compared to more complex interventions (i.e., control of arousal level and the use of cue words). Similarly, another type of intervention that has been shown to aid performance under pressure is ‘quiet eye’ training (e.g. Vine & Wilson, 2011; Vine, Moore, & Wilson, 2011). The quiet eye (QE) period is the duration of the final ocular fixation on a target before the initiation of movement (Vickers, 1996). QE training commonly involves the lengthening of the duration and improved consistency of this QE period. It has previously been surmised that the QE duration reflects a crucial period of cognitive processing where parameters of movement such as force, direction, and velocity are pre-programmed (Williams, Singer, & Frehlich, 2002). According to this viewpoint, longer QE durations in anxious conditions are related to improved pre-planning and ultimately improved performance. Of course, given the proposals of Englert and Bertram (2015), it is feasible to suggest that the aforementioned interventions would only be successful if the performer has sufficient self control strength to release the self evoked resources needed to inhibit state anxiety related attentional shifts and focus on the goal of improving planning processes.

In relation to ACT (Eysenck et al., 2007), the manipulation of pressure to perform results in shorter QE durations (reflective of impaired goal directed attentional control) and greater fixations of shorter duration (reflective of a stimulus driven attentional control system) when compared to non-pressured conditions (Wilson et al. 2009a, 2009b). These visual gaze measures offer support for Esyenck et al’s (2007) ACT over that of Masters (1992) CPH when explaining the pressure–performance relationship. However, in visual aiming tasks comparable to those of the current study, researchers have revealed that eye saccade distances and hand movement distances are closely coupled (Khan, Fourkas, Franks, Buckloz, & Hardy, 2002), that the eye doesn’t typically fixate on the target in goal directed aiming until movement initiation or relatively early in the movement trajectory (Abrams, Meyer, & Kornblum, 1990), and that tasks can be performed accurately under conditions of no vision (Khan et al. 2003a, 2003b). As such, the benefits of the typical QE effect observed in the complex, gross movement higher order tasks adopted by QE researchers (e.g., Vickers, 1996; Vine & Wilson, 2011; Vine et al., 2011; Wilson et al., 2009a, 2009b) may not transfer to the relatively simple and constrained video amplitude task of the current study. A paradigm that allows more complex tasks to be performed while still examining the effects of pressure on pre-planning and online control would help remedy this transfer limitation. Future research could then seek to concurrently examine QE duration along with pre-planning and online control processes under pressure conditions. This would allow investigation into an empirically linked relationship between longer QE, improved pre-planning, and improved performance.

### Potential limitations

Because our *pressure and online v offline visual aiming performance* research question is arguable the first of its kind, we chose not to conduct an a priori GPower analysis. The rationale being that whilst we state theoretically driven directional hypotheses, we did not have specific predictions regarding the size of the mean difference or associated standard deviations; basically because there was no previous research from which to speculate these values. As such, we adopted an approach of selecting the sample size for the current study based on those reported in previous *visual aiming* research that has utilised similar goal-directed aiming tasks (see Khan & Lawrence, 2005; Khan, Lawrence, Franks, & Buckloz, 2004; Khan et al. 2003a, 2003b; Khan, Sarteep, Mottram, Lawrence, & Adam, 2011; Lawrence, Khan, Buckloz, & Oldham, 2006; Lawrence et al., 2012b). Because our sample size of 24 is comparable to those of this previous research (average *n* = 17) we are reasonably comfortable with our confidence of the significance of the present findings. Furthermore, our error values for the control group are also comparable to those reported in the previous visual aiming research. To add further support to the *power* of the significance of our findings, the statistically significant observations between the control and experimental groups were in the theoretically predicted directions [these predictions were based on two well established and thoroughly researched theories; CPH (Masters, 1992) and ACT (Eysenck et al., 2007)]. However, we strongly recommend that researchers strive to utilise the findings of the current study to perform GPower analysis when determining sample sizes required for future research.

Whilst it is beyond the primary focus of the current research, there is little doubt that individual differences and personality play a significant role in the pressure–performance relationship. That is, whilst not an exhaustive list, it has been shown that trait anxiety (Horikawa & Yagi, 2012) and trait emotional intelligence (Laborde, Lautenbach, Allen, Herbert, & Achtzehn, 2014), affect the interaction between pressure and performance. For example, those individuals that demonstrate high levels of trait anxiety often report higher levels of state anxiety under pressure manipulations in comparison to their low trait anxiety counterparts (Horikawa & Yagi, 2012). Given the predictions of ACT Eysenck et al. (2007) and Englert and Bertrams (2015) proposed interaction between ACT and the strength model of self control, this trait-state anxiety relationship would likely result in more frequent observations of pressure related performance decrements in individuals with high levels of trait anxiety (see Horikawa & Yagi, 2012). Whilst the current study employed a randomised sampling paradigm when determining the sample, it is not possible to completely rule out the prospect that results were influenced by participant’s levels of trait anxiety (or any other personality trait). With this in mind, future research may wish to routinely include personality measures when conducting research aimed at investigating the pressure–performance relationship.

## Conclusion

The present study aimed to concurrently examine the effects of pressure on movement pre-planning and online control, both early and late in learning. Early in learning, performance in pressure conditions was comparable to a control group. Changes in the kinematic profile indicated that this effect was caused by both groups adopting similar strategies to control both the planning and the mechanics of the movement during the motor output. Late in learning; however, pressure resulted in a decrease in the use of online adjustments for movement control, but an increase in performance associated with more effective movement pre-planning. Recent research (Lawrence et al., 2012b) has revealed an inability to utilise online control processes during pressure conditions and we observe a similar finding in the present experiment. Thus, we conclude that participants consciously adopted a strategy of increasing effort, and thus the accuracy of the cognitive control processes associated with pre-planning, in an attempt to reduce the performance decrements associated with an inability to effectively use online control processes when performing under pressure.

## Footnotes

1 The point score was a direct measure of performance and was calculated using a combination of the absolute error for the criterion movement time and the end point error of the cursor. In other words, a combination of how close the participant was to meeting the 400 ms MT and how close their movement finished in relation to the target. A maximum of five points were possible for each component, meaning a maximum of 10 points were possible on any one trial. The maximum score of 10 was achieved if MT fell within ±10 ms of the criterion MT and cursor error fell within ±5 mm of the criterion target distance. These points reduced by one whole integer for every additional ±10 ms and every additional ±5 mm that the cursor fell outside of the criterion MT and the criterion target distance, respectively. For example, a trial with a MT of 379 ms and an error of 6 mm would be awarded be a total of 7 points. 3 points awarded for the MT falling ±21 ms outside the criterion MT and 4 points for the error falling ±6 mm outside of the criterion target distance.

2 It was hoped that pairing people with a bogus 'partner' would increase performance pressure and maintain engagement in the task (Beilock & Carr, 2001; Lawrence et al., 2012a, 2012b).

3 The rationale for monitoring mental effort was in line with the proposals within ACT (Esyenck et al., 2007). That is, one proposal of ACT is that if additional processing resources are available, then performance is less likely to be impaired by the presence of pressure. However, whilst the specifics of this additional resource and its processes have yet to be explicitly defined (Englert & Bertrams, 2015), mental effort was adopted as a measure of this self-regulatory process in line with previous research (see Lawrence et al., 2012a, 2012b; Lawrence et al., 2014; Oudejans & Pijpers, 2010; Nieuwenhuys and Oudejans, 2012).
